# Clinicopathologic Analysis and Prognostic Factors for Survival in Patients with Operable Ampullary Carcinoma: A Multi-Institutional Retrospective Experience

**DOI:** 10.3390/medicina60050818

**Published:** 2024-05-16

**Authors:** Nebi Serkan Demirci, Eyyup Cavdar, Nuriye Yildirim Ozdemir, Sinemis Yuksel, Yakup Iriagac, Gokmen Umut Erdem, Hatice Odabas, Ilhan Hacibekiroglu, Mustafa Karaagac, Mahmut Ucar, Banu Ozturk, Yakup Bozkaya

**Affiliations:** 1 Department of Medical Oncology, Cerrahpasa Faculty of Medicine, Istanbul University-Cerrahpasa, 34098 Istanbul, Türkiye; 2 Department of Medical Oncology, Adiyaman Training and Research Hospital, Adiyaman University, 02000 Adiyaman, Türkiye; 3 Department of Medical Oncology, Faculty of Medicine, Yıldırım Beyazıt University, 06010 Ankara, Türkiye; 4 Department of Medical Oncology, Dr. Lutfi Kirdar Kartal Education and Research Hospital, 34865 Istanbul, Türkiye; 5 Department of Medical Oncology, Balikesir Ataturk City Hospital, 10100 Balikesir, Türkiye; 6 Department of Medical Oncology, Faculty of Medicine, Sakarya University, 54050 Sakarya, Türkiye; 7 Department of Medical Oncology, Meram Medical Faculty, Necmettin Erbakan University, 42090 Konya, Türkiye; mustafakaraagac55@hotmail.com; 8 Department of Medical Oncology, Faculty of Medicine, Erciyes University, 38039 Kayseri, Türkiye; 9 Department of Medical Oncology, Akdeniz University, 07058 Antalya, Türkiye; drbanutr@yahoo.com; 10 Department of Medical Oncology, Yeniyuzyil University-Gaziosmanpasa Hospital, 34098 Istanbul, Türkiye; dr_yakupbozkaya@hotmail.com

**Keywords:** prognostic, ampullary carcinoma, survival

## Abstract

*Background and Objectives:* In ampullary cancer, 5-year survival rates are 30–50%, even with optimal resection and perioperative systemic therapies. We sought to determine the important clinicopathological features and adjuvant treatments in terms of the prognosis of patients with operable-stage ampullary carcinomas. *Materials and Methods:* We included 197 patients who underwent pancreaticoduodenectomy to treat ampullary carcinomas between December 2003 and May 2019. Demographics, clinical features, treatments, and outcomes/survival were analyzed. *Results:* The median disease-free survival (mDFS) and median overall survival (mOS) were 40.9 vs. 63.4 months, respectively. The mDFS was significantly lower in patients with lymphovascular invasion (*p* < 0.001) and lymph node involvement (*p* = 0.027). Potential predictors of decreased OS on univariate analysis included age ≥ 50 years (*p* = 0.045), poor performance status (*p* = 0.048), weight loss (*p* = 0.045), T3–T4 tumors (*p* = 0.018), surgical margin positivity (*p* = 0.01), lymph node involvement (*p* = 0.001), lymphovascular invasion (*p* < 0.001), perineural invasion (*p* = 0.007), and poor histological grade (*p* = 0.042). For the multivariate analysis, only nodal status (hazard ratio [HR]1.98; 95% confidence interval [CI], 1.08–3.65; *p* = 0.027) and surgical margin status (HR 2.61; 95% CI, 1.09–6.24; *p* = 0.03) were associated with OS. *Conclusions:* Nodal status and a positive surgical margin were independent predictors of a poor mOS for patients with ampullary carcinomas. Additional studies are required to explore the role of adjuvant therapy in patients with ampullary carcinomas.

## 1. Introduction

Periampullary tumors are neoplasms arising in close proximity to the papillary ampulla of the duodenum but can also originate in the distal regions of the pancreatic and common bile ducts. Although ampullary carcinomas (ACs) comprise only 0.2% of all gastrointestinal malignancies, they are the second most common periampullary tumors following pancreatic carcinomas of the head [1,2]. Primary ACs are extremely rare, with an incidence of approximately four to six cases per million but have tended to increase in the last three decades [3,4,5]. They cause 20% of malignant obstructions of the bile passage [5]. The most common histopathological type is adenocarcinoma, followed (in order) by neuroendocrine tumors and lymphoma [3].

Ampullary carcinomas are associated with relatively good prognoses compared to those of pancreatic and biliary carcinomas, given their high resection rates (90% in a recent series) [6]. The survival rates after AC resection are 30–50% at 5 years but less than 10% at 2 years for pancreatic carcinomas [7,8,9,10]. The main curative approach is resection with negative margins; the optimal management in terms of adjuvant treatment is controversial [11]. Surgeons also perform local excisions to avoid mortality and morbidity caused by major resections. Although the method remains very controversial, transduodenal excision can serve as a treatment option in selected patient groups (e.g., older patients, those with other comorbid conditions, patients with tumors < 2 cm in diameter, and those with polypoid tumors) [12]. Unfortunately, this procedure increases the risk of positive margins and, therefore, local recurrence. Also, local lymph node dissection is not performed when local resection is chosen, but T1 tumors may be associated with lymph node metastasis [13,14].

Despite the different prognoses, adjuvant treatment generally views the tumors as pancreatic carcinomas, not small intestinal cancers [15,16]. In the current study, we sought to define significantly prognostic clinicopathological factors and useful adjuvant treatment strategies for resected-stage ACs.

## 2. Materials and Methods

### 2.1. Patients

We interrogated a retrospective database and enrolled 197 patients with histologically proven non-metastatic ACs treated in seven oncology departments in Türkiye between December 2003 and May 2019. All underwent curative intent pancreaticoduodenectomy, including R0 and R1 resections. All were postoperatively monitored using chest radiography and computed tomography; routine blood tests and tumor marker assays were performed during follow up. Patients were followedup regularly every 6 months. This study was approved by our institute’s Ethics Committee.

### 2.2. Clinical and Histopathologic Features

We collected patient gender, age at diagnosis, performance status, and disease characteristics [stage, nodal status, degree of differentiation, and lymphovascular (LVI) and perineural invasion (PNI) status]. Stage and performance status were defined using the American Joint Committee on Cancer staging system (AJCC, 8th edition of the TNM classification) and the Eastern Cooperative Oncology Group (ECOG), respectively. Data on adjuvant treatments, like chemotherapy or chemoradiotherapy, were recorded in detail. Recurrence and survival information were obtained from medical charts and the governmental civil registry.

### 2.3. Statistics

All data were evaluated using the Statistical Package for Statistical Sciences (SPSS 26.0) for Windows 18. We obtained Kaplan–Meier estimates of overall and disease-free survival (OS and DFS) with 95% confidence intervals (CIs). OS was the time from the 1st day after surgery to death from any cause and DFS the time from the 1st day after surgery to the date of first recurrence (local or systemic). Univariate Cox regression was used to estimate hazard ratios (HRs) and their 95% CIs for OS and DFS. Variables with *p*-values < 0.1 or those that were clinically significant on univariate Cox regression analysis were subjected to multivariate analysis using stepwise selection The ratios of individuals surviving for up to 3 and 5 years were calculated using life tables. HRs with 95% CIs were calculated employing Cox’s proportional hazards model. The distributions of prognostic variables among treatment groups were compared using the chi-squared test. *p*-values < 0.05 were considered statistically significant.

## 3. Results

### 3.1. Demographic Data

Patient characteristics are shown in Table 1. The median age at diagnosis was 60 years (min-max, 29–90 years) with a slight male (60.9%) predominance. ECOG performance status was 0–1 in 78% of patients. Most (66%) presented with TNM stage 2 and 3 disease and half with nodal involvement (45.6%). The surgical margins were positive in 6.6% of patients. The LVI and PNI rates were 35% and 25.3% respectively. The vast majority (86.2%) exhibited adenocarcinoma histology. Approximately 1/3 of the patients did not receive adjuvant therapy (29.9%), 1/3 received only chemotherapy (CT) (37.6%), and the remaining 1/3 received chemoradiotherapy (CRT) (32.5%). In all patients who received adjuvant therapy, the agents were gemcitabine or fluoropyrimidine in varying proportions.

### 3.2. Recurrence and Life Data

At a median follow up of 32.1 months (min-max, 1.6–186.1 months), the median DFS (mDFS) and OS (mOS) were 40.9 (95% CI, 24.59–57.34 months) and 63.4 (95% CI, 24.56–102.31 months), respectively. Age ≥ 50 years (*p* = 0.045), ECOG performance status ≥ 2 (*p* = 0.048), nodal involvement (*p* = 0.001), advanced histological grade *(p* = 0.042), higher T stage (*p* = 0.018), LVI (*p* < 0.001), and PNI (*p* = 0.007) were found to be related to a poor mOS in the univariate analysis. In addition, stage 2 and 3 disease tended to be associated with a poorer mOS (*p* = 0.06) (Table 2). For the univariate analysis, the mDFS was significantly worse in patients with LVI (*p* < 0.001) and lymph node involvement (*p* = 0.027). Surgical margin positivity (*p* = 0.058) also tended to be associated with a poor mDFS. In our study, 60% of patients had jaundice at the time of diagnosis, but this had no effect on survival. (HR, 0.95; 95% CI, 0.61–1.49; *p* = 0.845)

**Figure 1:**
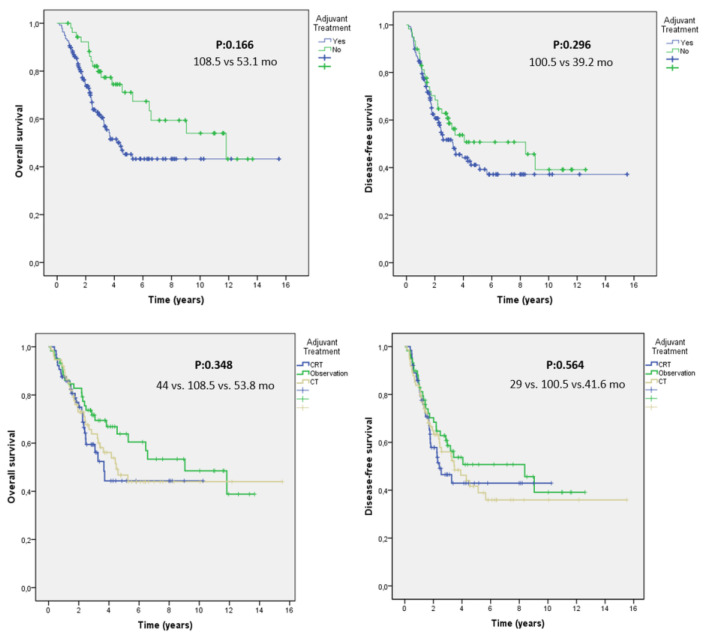
Kaplan–Meier curves comparing survival among patients who received adjuvant chemoradiation and chemotherapy and those treated with surgery alone. Adjuvant therapy was clinically significant but not statistically insignificantly associated with worse overall survival (*p* = 0.166) and was not significantly associated with disease-free survival (*p* = 0.296) in the univariate analysis.

There were statistically insignificant but clinically significant mOS and mDFS differences between cohorts who did and did not receive adjuvant treatment (Table 2 and Figure 1). Most patients with advanced T stages who were lymph node positive (83%, *p* < 0.001) and had LVI (78.3%, *p* = 0.052) and positive surgical margins (76.9%, *p* = 0.180) received adjuvant treatment.

**Figure 2:**
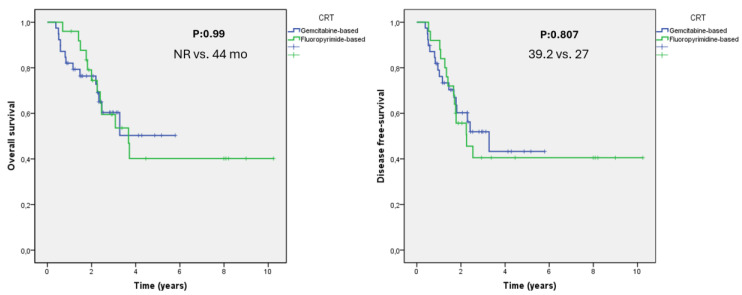
Kaplan–Meier curves comparing survival among patients who received adjuvant chemoradiation. The median disease-free survival times for fluoropyrimidine was 27 months and 39.2 months for gemcitabine (*p* = 0.807). The median overall survival time for fluoropyrimidine was 44 months and was NR for gemcitabine (*p* = 0.99). Abbreviation: NR, not reached.

The mOS was 53.8 months in the adjuvant CT group and 44 months in the CRT group compared to a median of 108.5 months in the observation group. There was no statistically significant mOS difference among the three arms (*p* = 0.348) (Table 2 and Figure 1). CRT did not afford a statistically significant mOS benefit but was clinically significant (44 vs. 108.5 months, *p* = 0.348), and this did not differ, regardless of whether gemcitabine or 5-fluorouracil was employed (*p* = 0.99) (Figure 1 and Figure 2). Although the mOS and mDFS did not differ significantly in statistical terms among patients with ampullary cancer treated or adjuvant therapy, clinically significant increases in OS (108.5 vs. 53.1 months, 95% CI, 34.70–71.54 months; *p* = 0.166) and DFS (100.5 vs. 39.2 months, 95% CI, 25.1–53.48 months; *p* = 0.296) were observed in patients who did not receive adjuvant therapy (Figure 1).

**Figure 3:**
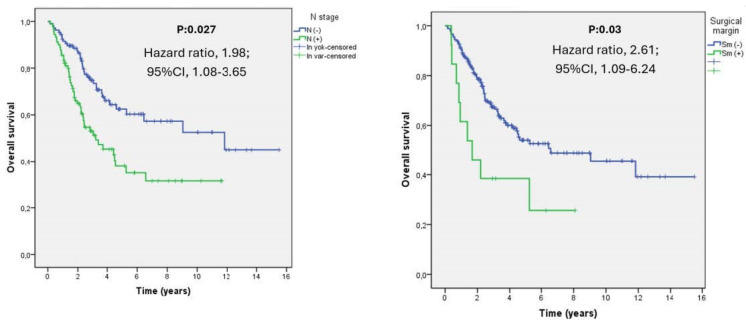
Kaplan–Meier curves for the multivariate analysis of overall survival. The results demonstrated that the only independent factors with a negative effect on the mOS were nodal metastasis and surgical margin positivity.

The multivariate Cox proportional hazard survival analyses of prognostic factors are shown in Table 3. The only independent factors negatively impacting the mOS were nodal metastasis (N stage; HR, 1.98, 95% CI, 1.08–3.65, *p* = 0.027) and surgical margin positivity (HR, 2.61, 95% CI, 1.09–6.24, *p* = 0.03) (Figure 3). Also, the advanced T stage was nearly significant (HR, 1.76, 95% CI, 0.96–3.2, *p* = 0.064).

## 4. Discussion

Pancreaticoduodenectomy (the Whipple procedure) remains the most important curative approach but has a high operative mortality rate of 15–23% [17,18]. At the time of diagnosis, 80% of patients are eligible for resection, but approximately half experience recurrences [19]. In an older series, those lacking adjuvant treatments, the figure was nearly 75% [20,21]. However, in a recent series, it was 12–40%, which was lower than our 49.7% in the surgery-only group [22,23]. Apart from suboptimal resection, subclinical nodal and regional disease are the main causes of frequent recurrence. Other factors that are prognostic of recurrence at diagnosis have also been defined [11,24,25,26,27]. Our rate of 47.9% is similar to those of other series.

Some researchers argue that AC patients have better prognoses because AC causes jaundice in the respectable stage of disease [28]. This may be true; obstructive jaundice is the most common presenting symptom (70–80% of patients) [2,29,30]. However, in other series, jaundice at diagnosis was found to be correlated with a worse prognosis [31,32,33]. Also, Kamisawa et al. found no correlation between survival and jaundice status in a series of 61 ampullary carcinoma patients [34]. In our study, 60% of patients had jaundice at the time of diagnosis, but this had no effect on survival. ECOG performance status at diagnosis can also be a valuable prognostic feature; this is the case for patients with a variety of solid tumors [35]. In our series, ECOG 2 and 3 status were associated with a worse prognosis.

Adenocarcinoma is the most frequent histological subtype of periampullary carcinomas [35,36]. We found that adenocarcinomas (85.9%), followed by mucinous carcinomas (9.5%), were the most common histological types. Previous data have indicated that (usually) poor tumor differentiation was associated with negative effects on survival [37,38,39]. In our series, poorly differentiated histology was indeed associated with worse survival. Several histopathologically prognostic factors have been reported to negatively affect the outcomes of patients with resected ampullary carcinomas. These include nodal metastasis, pancreatic invasion, PNI and LVI, positive surgical margins, advanced tumor grade, and a higher T stage [40,41,42,43,44]. Not all were identified in all series. The most important factor in terms of life expectancy is lymph node status. Metastasis in the nodal area is strongly associated with poor survival [42,43,45,46,47,48]. Roder et al. were the first to report that nodal metastasis was a negative prognostic factor [46]. Kim et al. showed that lymph node positivity was an independent (negative) predictive factor [49]. Various series reported lymph node positivity in 10 to 46% of patients, similar to our result (45.6%) [4,39,42]. Recent series reported median and 5-year survival rates of 16–24 months and 0–50% in lymph node-positive patients and 39–101.8 months and 39–81% in lymph node-negative patients [4,42,45,46,47]. We found a statistically significant mOS difference between the node-positive and -negative groups.

Lymph node status is associated with tumor size, grade, PNI, LVI, and T stage [39,46]. In a series of 450 patients treated at Johns Hopkins Hospital, nodal metastasis was directly related to the T stage; the nodal involvement percentage increased as the T stage rose [39]. Similar results were found in the present study; lymph node metastasis was more common in patients with stage T3–T4 than T1–T2 tumors. Outcomes are poor if the pancreas is involved. In a study from the Mayo Clinic, a statistically significant OS difference was apparent between those with stage T1–2 and T3–4 tumors [18]. About half of our cohort exhibited pancreatic involvement; our findings confirmed the relationship between pancreatic invasion and poor outcomes.

Most studies have shown that PNI adversely affects overall survival, but conflicting results have been reported [43,50,51,52]. Ghazzaway et al. found no correlation between PNI and survival in a series of 123 patients [53]. We observed that PNI negatively affected overall survival. LVI is a predictor of poor prognosis in patients with ampullary carcinomas [54]. Not surprisingly, in the present study, patients with LVI exhibited a shorter mOS and mDFS. Negative surgical margins correlated positively with better outcomes [4,54,55]; our surgically positive margin rate was similar to those of other series (3–9%) with lower mOS rates.

Although pancreaticoduodenectomy is potentially curative for patients with ampullary carcinomas, approximately half die from recurrences. Adjuvant strategies are required, but no consensus treatment is yet available because of the rarity of the disease and the lack of data on the benefits afforded by postoperative CT or CRT [15,16,56,57,58]. The ESPAC-3 trial enrolled principally ampullary carcinoma patients and sought to define the need for and the optimal type of adjuvant treatment. On subgroup analysis, the mOS values were 40.6, 57.8, and 70.8 months for the observation, FUFA (5-fluorouracil plus folinic acid), and gemcitabine arms, respectively, in those with ampullary carcinomas, irrespective of histology [56]. In our study, the mOS was 53.8 months in the adjuvant CT group and 44 months in the adjuvant CRT group compared to a median of 108.5 months in the observation group. There were no statistically significant mOS differences among the three arms. Further, local treatments are also controversial. In a series of 125 patients treated at the Mayo Clinic, the 5-year survival was 48% in a CRT group receiving FUFA compared to 11% in a no-treatment group. It was concluded that CRT significantly prolonged the mOS in patients with nodal metastasis [22]. In a joint study conducted by the Mayo Clinic and Johns Hopkins Hospital, no significant OS difference was apparent between an adjuvant CRT and a no-treatment group [18]. However, in a subgroup analysis of lymph node-positive patients, CRT was associated with a statistically significant OS difference. In one recent study, adjuvant therapy did not affect the mOS or mDFS [59]. We also found no statistically significant mOS benefits. Although there were no statistically significant differences in the OS or DFS among patients with ampullary cancer who received adjuvant therapy and those who did not, clinically significant increases in OS and DFS were observed in patients who did not receive adjuvant therapy. The shorter survival of patients receiving adjuvant treatment probably reflects the fact that patients on adjuvant therapy were more likely to be of a higher T stage, to be lymph node positive, to exhibit LVI, and to have more poorly differentiated tumors than others.

The AJCC staging systems for ampullary carcinoma do not consider many factors that affect prognosis, such as tumor differentiation and age. The systems predict outcomes in populations rather than in individuals. In a recent study, Li et al. developed and validated an accurate nomogram that predicted tumor recurrence probability in patients with non-metastatic ampullary carcinomas after surgical treatment [60]. The accuracy of the nomogram in terms of predicting disease-specific survival was 0.70 (95% CI 0.68–0.72), better than that of the AJCC staging systems [0.64 (95% CI 0.62–0.66; *p* < 0.001]. The nomogram should be used to help clinicians and patients assess the recurrence risk and choose appropriate adjuvant treatment.

The limitations of our study are that it was both retrospective and multicenter in nature. Also, subgroups with histological pancreaticobiliary and intestinal cancers were not distinguished. Thus, any differences in the survival of such subgroups could not be determined. Some histological grade and LVI data were missing. This may bias the results. However, we evaluated a large number of patients with a rare disease; we examined many clinicopathological features and the role of adjuvant therapy.

## 5. Conclusions

We suggest that ampullary carcinomas (especially the intestinal type) should be viewed as distinct from pancreatic carcinomas because, histologically, the former mimic intestinal tumors and survival is better than that associated with pancreatic carcinomas. Patients on adjuvant therapy exhibited poorer survival, probably because such patients exhibited worse clinicopathological features than others. Nodal and surgical margin status were independent predictors of the mOS in patients with carcinomas of the ampulla of Vater. Therefore, randomized multicenter studies that specifically address carcinoma of the ampulla of Vater are required to define the best treatment strategy for patients with nodal involvement and positive surgical margins.

## Figures and Tables

**Table 1:** Baseline patient demographics and clinical characteristics.

Characteristics	No. of Patients, *n* = 197 (%)
Age, years	
Median	60
Range	29–90
50	39 (19.8)
≥50	158(80.2)
Sex	
Mele	120 (60.9)
Female	77 (39.1)
Performance status	
0–1	154 (78.1)
2–3	43 (21.9)
TNM stage	
I	67 (34)
II	103 (52.2)
III	27 (13.8)
T stage	
1–2	99 (50.2)
3–4	98 (49.8)
N stage	
Positive	90 (45.6)
Negative	107 (54.4)
Histological grade	
Well differentiated	58 (29.4)
Moderately differentiated	75 (38)
Poorly differentiated	17 (8.6)
Pathological subtype	
Adenocarcinoma	170 (86.2)
Mucinous	18 (9.1)
Other	9 (4.6)
Lymphovascular invasion	
Yes	69 (35)
No	101 (51.2)
Perineural invasion	
Yes	50 (25.3)
No	120 (60.9)
Surgical margin	
Positive	13 (6.5)
Negative	184 (93.5)
Adjuvant treatment	
CT	74 (37.5)
Gemcitabine	43
Fluoropyrimidine	31
CRT	64 (32.4)
Gemcitabine	39
Fluoropyrimidine	25
Observation	59 (29.9)
Jaundice at diagnosis	
Yes	119 (60.5)
No	78 (39.5)

Abbreviations: CT, chemotherapy; CRT, chemoradiotherapy.

**Table 2:** Univariate survival analysis according to clinicopathological parameters.

Patients	*n* (%)	3-Year Survival, %	5-Year Survival, %	mOS (mo)	*p*	mDFS (mo)	*p*
All patient groups	197	65	51	63.4		40.9	
Age, years							
<50	39 (19.8)	91	66	78.8	0.045	54.3	0.270
≥50	158 (80.2)	58	48	53.1		30.5	
Performance status							
0–1	154 (78.2)	66	55	108.5	0.048	41.6	0.1
2–3	43 (21.8)	59	37	46		28.4	
Nodal involvement							
Positive	90 (45.6)	53	32	38.8	0.001	28.4	0.027
Negative	107 (54.4)	75	62	141.9		67.8	
Histological grade							
Well differentiated	58 (29.4)	71	61	NR	0.042	54.3	0.082
Moderately differentiated	75 (38)	58	51	63.1		38.3	
Poorly differentiated	17 (8.6)	51	26	36.9		26.9	
Lymphovascular invasion							
Yes	69 (35)	51	37	36.9	<0.001	27	<0.001
No	101 (51.2)	75	66	NR		108.5	
Perineural invasion							
Yes	50 (25.3)	55	39	39.2	0.007	38.8	0.089
No	120 (60.9)	70	61	108.8		67.8	
T stage							
1–2	99 (50.2)	73	59	108.5	0.018	54.3	0.116
3–4	98 (49.8)	56	42	39.3		30.6	
TNM stage							
I	67 (34)	75	64	108.5	0.06	67.8	0.192
II	103 (52.2)	61	47	53.1		34.4	
III	27 (13.8)	55	36	38.8		26.3	
Surgical margin							
Negative	184 (93.5)	67	54	20.1	0.01	41.6	0.058
Positive	13 (6.6)	38	38	78.8		15.5	
Adjuvant treatment							
None	59 (29.9)	71	71	108.5	0.348	100.5	0.564
CRT	64 (32.5)	58	44	44		29	
CT	74 (37.6)	64	46	53.8		41.6	

Abbreviations: CT, chemotherapy; CRT, chemoradiotherapy; NR, not reached.

**Table 3:** Multivariate Cox proportional hazard survival analysis of prognostic factors.

Factors	Relative Risk	95% CI	*p*-Value
Nodal Status			
Positive	1.98	1.08–3.65	0.027
Negative			
Surgical Margin			
Positive	2.61	1.09–6.24	0.03
Negative			
Tumor Stage			
T1–T2	1.76	0.96–3.2	0.064
T3–T4			

## Data Availability

The data sets used in the present study are available from the corresponding author upon reasonable request.
